# Lung Cancer Diagnosis Based on an ANN Optimized by Improved TEO Algorithm

**DOI:** 10.1155/2021/6078524

**Published:** 2021-07-16

**Authors:** Rong Shan, Tahereh Rezaei

**Affiliations:** ^1^College of Computer, Weinan Normal University, Weinan, Shaanxi, China; ^2^Neuroscience Research Center, Shiraz, Iran

## Abstract

A quarter of all cancer deaths are due to lung cancer. Studies show that early diagnosis and treatment of this disease are the most effective way to increase patient life expectancy. In this paper, automatic and optimized computer-aided detection is proposed for lung cancer. The method first applies a preprocessing step for normalizing and denoising the input images. Afterward, Kapur entropy maximization is performed along with mathematical morphology to lung area segmentation. Afterward, 19 GLCM features are extracted from the segmented images for the final evaluations. The higher priority images are then selected for decreasing the system complexity. The feature selection is based on a new optimization design, called Improved Thermal Exchange Optimization (ITEO), which is designed to improve the accuracy and convergence abilities. The images are finally classified into healthy or cancerous cases based on an optimized artificial neural network by ITEO. Simulation is compared with some well-known approaches and the results showed the superiority of the suggested method. The results showed that the proposed method with 92.27% accuracy provides the highest value among the compared methods.

## 1. Introduction

**Figure 1:**
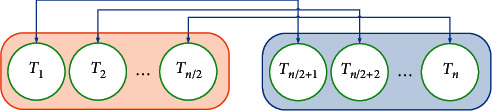
The heat transfer groups and the environment and cooling objects pairs.

The proliferation of lung diseases in today's industrialized societies doubles the need for modern methods of accurate and early diagnosis. Among lung diseases, lung cancer is still recognized as one of the most dangerous cancers. Cancer means the abnormal growth, and sometimes proliferation of cells in the body. All cancers have an uncontrolled growth pattern and a tendency to detach from the source and metastasize [1]. A normal lung cell may become a lung cancer cell for no apparent reason, but in most cases, the transformation is the result of repeated exposure to carcinogens such as alcohol and tobacco. The appearance and function of cancer cells are different from normal cells. A mutation or change in the DNA or genetic material of a cell occurs [2]. DNA is responsible for controlling the appearance and function of cells. When a cell's DNA changes, that cell differentiates from the healthy cells next to it and no longer does the body's normal cells. This altered cell separates from its neighboring cells and does not know when it should stop growing and die [3]. In other words, the altered cell does not follow the internal commands and signals that other cells are in control of and acts arbitrarily instead of coordinating with other cells [4]. One-third of all cancer deaths are due to lung cancer. About 80% of patients have five years left in their best condition after being diagnosed with this type of cancer [5]. Based on a survey by the American Cancer Society (ACS), lung cancer in both men and women is the second most prevalent cancer in the United States [6]. Approximately 228,820 new cases and approximately 135,720 lung cancer deaths, based on ACS figures, will occur [5].  Figure 1 shows the statistical information for the lead cancers and their death number in 2019 based on the American Cancer Society of lung cancer screening [7].

Air pollution due to the industrialization of cities, tobacco use, and genetic factors are the main causes of these diseases [8]. Early diagnosis of lung disease will have a major impact on the possibility of definitive treatment of the disease. Major diagnostic methods for lung cancer include radiographic imaging and computed tomography, biopsy, bronchoscopy, and examination of cells in the sputum. Meanwhile, the CT scan imaging method is widely used as a superior diagnostic method. In this diagnostic method, the doctor examines possible nodules on the images. A pulmonary nodule is a small, round, opaque mass that forms inside the lung tissue [9, 10]. In other words, nodules are spherical radiographic opacities of less than three centimeters in diameter.

Formerly, lung diseases were diagnosed based on the help of experts' eye ability with no use for computer science. However, recently based on the different imaging techniques based on computer science and artificial intelligence, the diagnosis can be more precise. In most of these methods, after capturing the images from the patient, different image processing methods have been performed for tumor diagnosis [11].

## 2. Image Preprocessing

The analyzed images for validation in this study are collected from the *Lung CT-Diagnosis database* which has been provided by the Cancer Imaging Archive [12]. This database contains a collection of publicly available medical images for different cancers [13] with contrast-enhanced CT scan images stored in the Dicom format. This database is collected from 61 patients such that 4682 different images have been acquired from them.

After image acquisition, the min-max normalization method is established for them to scale the acquired images between 0 and 1. This study uses 250 × 250 scale normalization for this purpose. By considering a grayscale image with *n* dimension which has the following limitation: *I*_*n*_=[*X*⊆*R*^*n*^]⟶[*a*,…, *b*], the normalized image, *I*^*∗*^, can be achieved by the following formulation:

(1) $$\begin{array}{c} I^{*}=a_{\text{new}}+\frac{b_{\text{new}}-a_{\text{new}}}{b-a}\times \left(I-a\right), \end{array}$$

where *I*^*∗*^=[*X*⊆*R*^*n*^]⟶[*a*_new_,…, *b*_new_], *a* and *b* describe the intensity values in the grayscale image, and *a*_new_ and *b*_new_ describe the intensity values for the normalized image [14].

Also, since the reason that all CT scan images have a kind of visual noise, they need a denoising filter to resolve the problem. A CT scan image faces different factors and includes different types, such as Gaussian noise, Shot Noise, Poisson Noise, Speckle Noise, and Salt and Pepper Noise [15]. Noise hides slight details of the CT scan image. This shows that we need a tool for noise removal before starting the CT scan images.

One of the popular noise reduction techniques for CT scan images is mean filtering. The definition of the mean (average) filter works on averaging any aspect of the picture to the neighbors [16]. The average filter calculates and divides the sum of all the pixels in the filter window by the total number of pixels [4]. It then replaces the value of the center pixel with the calculated average. The result value for each indexed pixel (*i*, *j*) is determined as follows:

(2) $$\begin{array}{c} R\left(i,j\right)=\frac{\left[I\left(i-1,j-1\right)+I\left(i-1,j\right)+I\left(i-1,j+1\right)+I\left(i,j-1\right)+I\left(i,j\right)+I\left(i,j+1\right)+I\left(i+1,j-1\right)+I\left(i+1,j\right)+I\left(i+1,j+1\right)\right]}{9}. \end{array}$$

## 3. Image Segmentation

One of the most important issues in image processing is the identification and separation of the image into its main components. Image segmentation determines the success or eventual failure of image analysis methods. And yet, due to its wide application, it has suitable research fields. The accuracy of this study in fields such as medicine is very important to preserve and protect human life. Thresholding is one of the most convenient methods for image segmentation [17]. By applying a thresholding method to a grayscale image, a binary image is obtained that delineates the boundaries of the objects in the image with appropriate accuracy.

The lower the threshold is, the more errors are detected and the more sensitive the results are to noise and unrelated image features [18]. On the other hand, a high threshold may miss weak errors or parts of errors. Since the main purpose of the fabric fault detection system is to find all possible faults, the threshold must be chosen to achieve this goal [19]. There are several methods for this practice in image processing science, and some separation methods are used for specific images [20]. One way to select the appropriate threshold is to use the trial-and-error method, in which different values of the threshold are selected and the image resulting from the application of this threshold is judged by the viewer. The simplest type of separation is called general separation, which is based on the image histogram. The input of this function is a gray image or a color image [21]. Its output is also a black and white image (binary). In this method, the threshold value at any point in the image is defined based on the local properties of the image in the neighborhood of that pixel. In this paper, Kapur thresholding has been used. Assume that the gray levels in an image with *N* pixels and *L* gray levels are in the range [0,1,…, *L* − 1]. Kapur method obtains the thresholds based on Kapur entropy maximization and based on the information obtained from the histogram of gray surfaces, which is defined as follows in the case of two-level segmentation (thresholding):

(3) $$\begin{array}{c} \text{Maximize }f_{1}\left(t\right)=H\left(0,t\right)+H\left(t,L\right), \end{array}$$

such that

(4) $$\begin{array}{c} H\left(0,t\right)=-\overset{t-1}{\underset{i=0}{\sum }}\frac{p_{i}}{w_{o}}\ln\frac{p_{i}}{w_{o}},\;w_{o}=\overset{t-1}{\underset{i=0}{\sum }}p_{i},\\ H\left(t,L\right)=-\overset{t-1}{\underset{i=0}{\sum }}\frac{p_{i}}{w_{i}}\ln\frac{p_{i}}{w_{i}},\;w_{i}=\overset{t-1}{\underset{i=0}{\sum }}p_{i}. \end{array}$$

The value of the optimal threshold point is the amount of gray area *t* that maximizes the function. For correct diagnosis of the tumors, the cancerous region should be detected with high precision. As mentioned before, image thresholding has been used for identifying the cancerous region. However, mathematical morphology is needed to better filter the detected areas based on image thresholding [22]. Mathematical morphology algorithm is a new technique for processing and analyzing signals and images. The basic idea of this technique is based on the analysis of geometric information by exploring an image with a small geometric pattern called a structuring element. This study uses three popular techniques including opening, closing, and filling holes. The first operator in the region filling is based on complementation, intersections, and dilation operators and is achieved by the following equation:

(5) $$\begin{array}{c} X_{k}=\left(X_{k-1}⊕B\right)\cap A^{c},\;k=1,2,3,\ldots , \end{array}$$

where *A* indicates a group of boundaries and *B* defines the organizing element. This operator will end if *X*_*k*_=*X*_*k*−1_.

The mathematical opening is the second utilized operator. The opening of element *A* with element *B* has been obtained with *A* erosion by structure element *B*, followed by a dilation of the resulted image by structure element *B*, that is,

(6) $$\begin{array}{c} A\circ B=\left(A⊖B\right)⊕B. \end{array}$$

The key goal of the mathematical opening is to remove the minor blemishes in the area that can be missed during the diagnosis of lung cancer.

Finally, the mathematical closing operator is used to smooth the counters, fuses narrow breaks, eliminates small holes, and fills gaps in the contour and long thin gulfs. This operation is formulated as follows:

(7) $$\begin{array}{c} A\cdot B=\left(A⊕B\right)⊖B. \end{array}$$

## 4. Features Extraction

The purpose of feature extraction is to make raw data more usable for future statistical processing. Feature extraction is a very common process in different types of data processing such as image processing and audio processing. Feature extraction means selecting a feature that can describe the image with little information. These features must have properties so that a set of these features is described uniquely in each image. If a set of these attributes are the same for two samples, then you will not be able to distinguish two samples with any classifier in the classification section.

The main reasons for extracting features from images are image simplification, reduced processing time and memory, and increased accuracy and efficiency. So, feature extraction is a process in which data is mapped in a high-dimension space to a lower dimension space. This mapping can be linear (such as principal component analysis) or nonlinear. How to select these features requires data properties to be examined, and to extract it, preprocessing operations and various filters must be applied to the image to turn the image into the desired information. In this study, GLCM features have been used for extracting the lung cancer images information.

The GLCM method is one of the most efficient techniques for extracting tissue from medical images. This matrix is a square matrix with dimensions *N* × *N* where *N* is the number of degrees of gray in the image. Each element of this matrix represents the number of pairs of pixels that have degrees of gray on the surface of the image and are spaced in a certain direction from each other and to a certain pixel distance. After calculating the matrix, different parameters of the image texture can be extracted from it. In this study, the mentioned technique was used to extract tissue in lung tumor images. In the following, the utilized features have been explained.

### 4.1. Contrast

The contrast regulates the intensity value of the pixel and its neighbor in the image. This feature is achieved by the following equation:

(8) $$\begin{array}{c} \text{Contrast}=\sum_{i=0}^{m-1}\sum_{j=0}^{n-1}{\left(i-j\right)}^{2}f\left(i,j\right). \end{array}$$

### 4.2. Correlation

The correlation feature describes the dependency on spatial features among the pixels. This feature can be mathematically given as follows:

(9) $$\begin{array}{c} \text{Cor}=\frac{\sum_{i=0}^{m-1}\sum_{j=0}^{n-1}\left(i,j\right)f\left(i,j\right)-\mu_{i}\mu_{j}}{\sigma_{i}\sigma_{j}}. \end{array}$$

### 4.3. Homogeneity

Homogeneity is a local uniformity feature that makes single/multiple intervals govern the textured and nontextured characteristics. This feature is achieved by the following:

(10) $$\begin{array}{c} H=\sum_{i=0}^{m-1}\sum_{j=0}^{n-1}\frac{1}{1+{\left(i-j\right)}^{2}}f\left(i,j\right). \end{array}$$

### 4.4. Energy

The energy feature regulates the number of repetitive pixel pairs. This feature is mathematically obtained by the following equation:

(11) $$\begin{array}{c} \text{Energy}=\sqrt{\sum_{i=0}^{m-1}\sum_{j=0}^{n-1}f^{2}\left(i,j\right)}. \end{array}$$

### 4.5. Entropy

The entropy is a feature that indicates the image selected interference based on the following equation:

(12) $$\begin{array}{c} \text{Ent}=-\sum_{i=0}^{m-1}\sum_{j=0}^{n-1}\log_{2}\;f\left(i,j\right). \end{array}$$

## 5. Features Selection

Some of the different extraction characteristics in the images are so important and crucial for classification. In the meantime, those features that contain information which is not notable can have high potential when combined with other features. Any of these attributes could also have no useful data at all. This shortcoming can be resolved by different works. In this paper, an optimization-based methodology has been proposed for this purpose. The optimization method for the features selection can be achieved by the following equation:

(13) $$\begin{array}{c} \text{FF}=\frac{\left(\text{TP}\times \text{TN}\right)-\left(\text{FP}\times \text{FN}\right)}{{\left(\left(\text{TN}+\text{FP}\right)\times \left(\text{TP}+\text{FP}\right)\times \left(\text{TP}+\text{FN}\right)\times \left(\text{TN}+\text{FN}\right)\right)}^{\left(1/2\right)}}, \end{array}$$

where TP signifies the true positive, TN describes the true negative, FN represents the false negative, and FP defines the false positive.

## 6. Improved Thermal Exchange Optimization Algorithm

In Thermal Exchange Optimization (TEO), the temperature of the objects indicates the individual position and with objects grouping, it is started to be exchanged. Therefore, new temperatures indicate their updated positions [23].

### 6.1. Newton's Law of Cooling

In the seventeenth century, the English scientist Isaac Newton studied the cooling of objects. The experiments showed that the cooling rate was approximately proportional to the temperature difference between the heated object and the environment. This fact is written as a differential relation:

(14) $$\begin{array}{c} \frac{\text{d}Q}{\text{d}t}=\alpha \times A\times \left(T_{s}-T_{b}\right), \end{array}$$

where *Q* describes the heat, *A* signifies the area of the body surface that transmits heat, *T*_*b*_ defines the body temperature, *T*_*s*_ represents the ambient temperature, and *α* determines the heat transfer coefficient which is dependent on the geometry of the object, surface state, heat transfer mode, and other factors.

The heat loss in time d*t* is *α* × *A* × (*T*_*s*_ − *T*)d*t* which defines the alteration in stored heat as the temperature falls d*T*, i.e.,

(15) $$\begin{array}{c} V\times \rho \times c\times \text{d}T=-\alpha \times A\times \left(T-T_{b}\right)\text{d}t, \end{array}$$

where *V* defines the volume (m^3^), *ρ* describes the density (kg/m^3^), and *c* signifies the specific heat (J/kg/K).

Therefore,

(16) $$\begin{array}{c} \frac{T-T_{b}}{T_{M}-T_{b}}=\exp\left(\frac{-\alpha \times A\times t}{V\times \rho \times c}\right), \end{array}$$

where *T*_*M*_ is the starting high temperature.

The above equation is valid when (*α* × *A* × *t*)/(*V* × *ρ* × *c*) is not a function of *T*, i.e.,

(17) $$\begin{array}{c} \gamma =\frac{\alpha \times A}{V\times \rho \times c}, \end{array}$$

where *γ* is constant. Therefore,

(18) $$\begin{array}{c} \frac{T-T_{b}}{T_{M}-T_{b}}=\exp\left(-\gamma t\right). \end{array}$$

And finally, the equation can be rewritten as follows:

(19) $$\begin{array}{c} T=\left(T_{M}-T_{b}\right)\times \;\exp\left(-\gamma t\right)+T_{b}. \end{array}$$

### 6.2. Inspiration

During the TEO algorithm, some individuals have been considered as the cooling objects and the residual individuals have been considered as the environment; then it has been in reverse. The method of simulation of the TEO algorithm is given in the following.

The first step is initialization. The initial temperature for all of the objects has been defined in an m-dimensional solution space as follows:

(20) $$\begin{array}{c} T_{i}^{0}=T_{\min}+\text{rand}\left(T_{\max}-T_{\min}\right), \end{array}$$

where *T*_*i*_^0^ signifies the initial solution vector of the object number *i*, rand describes a random vector with components in the range [0,1], *T*_min_ and *T*_max_ describe the minimum and the maximum limitations for the decision variables.

After initializing the objects, the value of the objective function for all of the individuals is evaluated. During the process, some historically best *T* vectors have been stored in a memory called Thermal Memory (TM) to use their position to develop the algorithm efficiency with no extra computational cost. After selecting some best values in TM, they have been added to the population and for the same numbers of them, worst individuals have been eliminated. Individuals have been divided into two equal groups.  Figure 1 shows this division. For example, *T*_1_ defines an environment object for *T*_(*n*/2)+1_ cooling object and contrariwise.

During the process, if an object has lower *γ*, the temperature exchanging has been established slowly. Therefore, the value of *γ* for the objects has been established based on the following equation:

(21) $$\begin{array}{c} \gamma =\frac{\text{Cos}\left(\text{object}\right)}{\text{Cos}\left(\text{worst object}\right)}. \end{array}$$

Time is another parameter that is important in this algorithm. This parameter is related to the iteration number. This parameter can be formulated by the following equation:

(22) $$\begin{array}{c} t=\frac{\text{iteration}}{\text{Max}\cdot \text{iteration}}. \end{array}$$

Generally, an important ability of the metaheuristics is to escape from the local optimum. During this process, the environmental temperature has been altered by the following equation:

(23) $$\begin{array}{c} T_{i}^{e}=\left(1-\left(c_{1}+c_{2}\times \left(1-t\right)\times \text{rand}\right)\right)\times T_{i}^{\prime e}, \end{array}$$

where *c*_1_ and *c*_2_ represent the control variables. *T*_*i*_^′*e*^ describes the object earlier temperature that has been modified to *T*_*i*_^*e*^.

Based on the previous models, the new temperatures of the objects have been updated by the following equation:

(24) $$\begin{array}{c} T_{i}^{+}=T_{i}^{e}+\left(T_{i}^{\text{old}}-T_{i}^{e}\right)\exp\left(-\gamma t\right). \end{array}$$

Another parameter in the algorithm is Pr with (0, 1) that stated whether a component in the cooling objects should be changed or not.

All of the Pr agents have been compared with *R*(*i*)(*i*=1,2,…, *n*) which is a randomly distributed value in the range [0, 1]. If *R*(*i*) < Pr, one dimension of the agent number *i* has been randomly chosen and the value is redeveloped by the following:

(25) $$\begin{array}{c} T_{i,j}=T_{j}^{\min}+\text{rnd }\left(T_{j}^{\max}-T_{j}^{\min}\right)\exp\left(-\gamma t\right), \end{array}$$

where *T*_*i*,*j*_ signifies the *j*^th^ variable of the *i*^th^ agent and *T*_*j*_^min^ and *T*_*j*_^max^ represent the lower and the upper bounds of the *j*^th^ variable, respectively. To keep the structures of the agents unchanged, just one dimension has been altered.

Finally, the stopping criteria are checked to terminate the algorithm in the considered criteria.

### 6.3. Improved Thermal Exchange Optimization (ITEO)

The basic Thermal Exchange Optimization algorithm suffers some disadvantages like stability and premature convergence problems. This case leads us to design a modified version of the TEO algorithm to refine these drawbacks as possible.

The first modification is to use Lévy flight (LF) as a proper mechanism. This mechanism has been commonly employed in metaheuristic algorithms to solve premature convergence shortcomings [24]. During this mechanism, a random walk policy has been utilized for proper adjusting of the local search that is mathematically represented as follows:

(26) $$\begin{array}{c} \text{Le}\left(x\right)\approx \frac{1}{x^{1+\tau }},\\ x=\frac{A}{{\left|B\right|}^{\left(1/\tau \right)}},\\ \sigma^{2}={\left\{\frac{\Gamma \left(1+\tau \right)}{\tau \Gamma \left(\left(1+\tau \right)/2\right)}\times \frac{\sin\left(\pi \tau /2\right)}{2^{\left(1+\tau \right)/2}}\right\}}^{\left(2/\tau \right)}, \end{array}$$

where *A* ~ *N*(0, *σ*^2^), *B* ~ *N*(0, *σ*^2^), *τ* signifies the Lévy index which is located in the range [0, 2] (here, *τ*=1.5 [25]), Γ(·) represents Gamma function, and *w* defines the step size.

By assuming the above equations, the updating formulation for the TEO algorithm is as follows:

(27) $$\begin{array}{c} T_{i,j^{\text{new}}}=T_{j}^{\min}+\text{Le }\left(T_{j}^{\max}-T_{j}^{\min}\right)\exp\left(-\gamma t\right). \end{array}$$

The second modification is to use the chaos mechanism for improving the system convergence speed. Here, we used Singer function for chaos modification [26, 27]. By considering this mechanism, rnd can be updated as follows:

(28) $$\begin{array}{c} {\text{rnd}}_{i+1}=1.07\left(7.9\times {\text{rnd}}_{i}-23.3\times {\text{rnd}}_{i}^{2}+28.7\times {\text{rnd}}_{i}^{3}-13.3\times {\text{rnd}}_{i}^{4}\right), \end{array}$$

where rnd_0_ ∈ [0,1].

### 6.4. Algorithm Authentication

The simulations are applied to a Core (TM) i7-4720HQ 1.60 GHz with 8 GB RAM under and simulations are applied to the MATLAB 2017b environment. To prove the effectiveness of the suggested ITEO algorithm, it is performed on some different benchmark functions, i.e., Ackley, Rastrigin, Sphere, and Rosenbrock, and the results have been compared with some well-known and new optimization techniques, i.e. Locust Swarm Optimization Algorithm (LSO) [28], Crow Search Algorithm (CSA) [29], Multi-Verse Optimizer (MVO) [30], search and rescue (SAR) algorithm [31], and the basic Thermal Exchange Optimization (TEO) [10].  Table 1 tabulates the parameter settings of the studied algorithms.

To clarify the studied benchmark functions, the equations and the boundaries have been given in Table 2. During the simulation, all of the algorithms run 40 times independently for all of the benchmark functions and the maximum iteration for all of the algorithms is considered 500.

Table 3 tabulates the results of the analysis of the algorithms on the benchmark functions based on four measurement indicators including minimum, maximum, mean, and standard deviation (std).

As can be observed from the results, the results of all four indicators based on the suggested ITEO have the minimum value. This indicates that the suggested algorithm has the minimum error ratio for all of the metrics. The minimum value of the indicator “minimum” shows that the proposed ITEO algorithm has the highest precision among the others. Plus, the minimum value of the “std” for the ITEO algorithm shows its higher consistency toward the other methods.

## 7. Classification

For the final diagnosis of the features, a classifier is needed. In this study, a new optimized version of Artificial Neural Network (ANN) has been used [33]. The ANN is an almost new methodology with high efficiency for different classification applications. The ANN makes a proper relationship between input and output data. The sensitivity of the ANN is so low toward errors [34]. This method can be used for the accurate classification of medical images by correct training with no mathematical modeling [35, 36]. A popular and simple technique among different types of ANN is the multilayer perceptron (MLP) neural network. An MLP is a mathematical model of a natural brain [34]. An MLP is a model with several numbers of weights and biases that are connected for brain performance modeling. The popular method for error minimization in MLPs is backpropagation (BP). In BP, the values of the weights and biases have been adjusted for minimizing the error between the output value and the desired value [37]. This method uses Gradient Descent (GD) algorithm for minimization. One significant problem of the GD algorithm is that they have been easily trapped into the local minimum.

The output of each layer in the network is as follows:

(29) $$\begin{array}{c} z_{i}=\sum_{j=1}^{n}w_{ij}\alpha_{i}+b_{j}, \end{array}$$

where *α*_*i*_ indicates the *i*^th^ input variable, *b*_*j*_ signifies the *j*^th^ bias for the neuron, and *w*_*ij*_ describes the relation weight between *α*_*i*_ and the *j*^th^ invisible neuron.

After that, the activation function has been performed to trigger the output of the neurons. This research employs sigmoid function for this purpose:

(30) $$\begin{array}{c} f_{j}\left(x\right)=\frac{1}{1+e^{-z_{j}}}. \end{array}$$

And the output layer gives

(31) $$\begin{array}{c} y_{i}=\sum_{j=1}^{m}w_{kj}\alpha_{i}+\beta_{k}. \end{array}$$

The ANN calculates mean square error (MSE) between the desired and the observed output, i.e.,

(32) $$\begin{array}{c} \text{MSE}=\frac{1}{n}\sum_{i=1}^{n}y_{i}-d_{i}, \end{array}$$

where *n* describes the number of the steps in the training data collection and *y*_*i*_ and *d*_*i*_ represent the observed value and the desired value, respectively.

**Figure 2:**
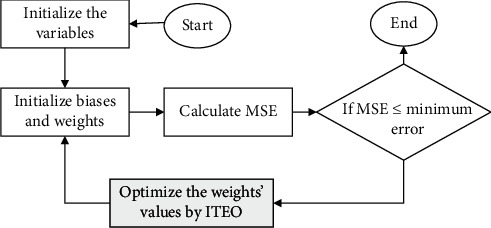
The flowchart of classification based on optimized ANN/ITEO.

As mentioned earlier, GD algorithm for minimizing the MSE has some problems. Therefore, here for minimizing the MSE and optimizing the classifier, the suggested Improved Thermal Exchange Optimization has been used.  Figure 2 shows the method of this idea.

## 8. Results and Discussion

**Figure 3:**
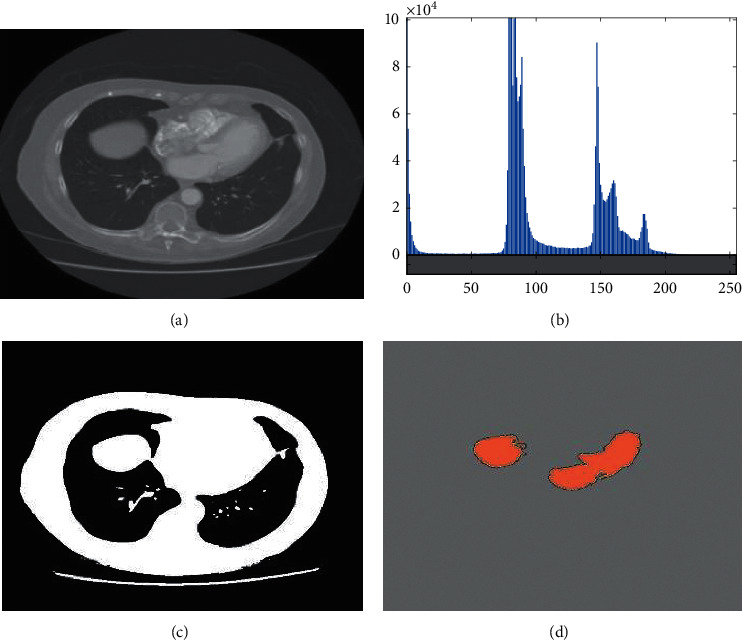
An example of the image segmentation: (a) Original image, (b) histogram of the image, (c) image segmentation based on Kapur technique, and (d) image (c) after mathematical morphology.

As mentioned before, the main idea of this study is to propose a pipeline methodology for lung cancer diagnosis. The method starts with a preprocessing method for enhancing the quality of the original image. After image preprocessing, a simple image thresholding has been done based on Kapur for lung areas. Afterward, optimal features have been selected from GLCM features based on an improved version of the Thermal Exchange Optimization algorithm. Finally, an optimized MLP system was based on the introduced Improved Thermal Exchange Optimization algorithm. The method has been validated based on the Lung CT-Diagnosis dataset collected by the Cancer Imaging Archive [12].  Figure 3 shows an example of the image segmentation.

### 8.1. Dataset Description

In this study, the Lung CT-Diagnosis database has been utilized. This database contains several images in Dicom format, employed from capturing 61 patients. The number of the total images is 4682. The method has been simulated based on MATLAB 2017b environment and performed to the database based on the following configuration: Corei7 laptop with 16 GB RAM and CPU@2.6 GHz processor.

### 8.2. Simulation Results

Table 4 indicates the GLCM data results of 20 first images from the Lung CT-Diagnosis database. As can be observed from Table 4, nineteen numbers of GLCM features are employed for the feature extraction (Table 5).

After feature selection based on the Improved Thermal Exchange Optimization, the optimum features based on the suggested ITEO methodology are evaluated and shown in Table 6.

The optimum threshold achieved by ITEO has been used to select the features. For the cost function, the best optimal value earned is 0.75. To test the final efficacy of the suggested technique, three measurement indicators including sensitivity, precision, and accuracy have been analyzed. The mathematical formulations of the indicators are given below:

(33) $$\begin{array}{c} \text{Accuracy}\left(\%\right)=\frac{\left(\text{TP}+\text{TN}\right)}{\left(\text{TP}+\text{FP}+\text{FN}+\text{TN}\right)},\\ \text{Specificity}\left(\%\right)=\frac{\text{TN}}{\left(\text{FP}+\text{TN}\right)},\\ \text{Sensitivity}\left(\%\right)=\frac{\text{TP}}{\left(\text{TP}+\text{FN}\right)}, \end{array}$$

where TN is truly negative, TP is truly positive, FN is false negative, and FP is false positive.

**Figure 4:**
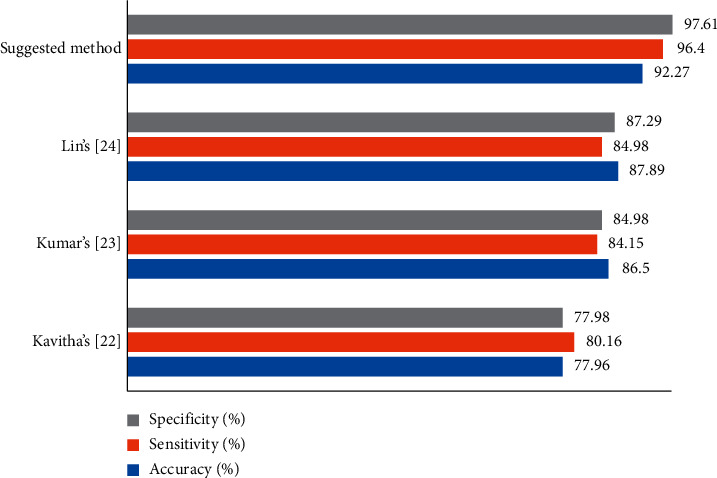
The efficiency validation of the suggested method compared with some well-known methods based on three mentioned indicators.

To verify the higher efficiency of the proposed method, a comparison analysis of the method has been applied toward some state-of-the-art algorithms, including Kavitha's [38], Kumar's [39], and Lin's [40], applied to the Lung CT-Diagnosis database. The comparison results are illustrated in a bar chart in Figure 4.

As can be observed from Figure 4, the proposed method has the best precision for the Lung CT-Diagnosis database and Lin's, Kumar's, and Kavitha's are placed in the next ranks. The results show also the proposed method.

## 9. Conclusions

The main purpose of this study is to propose an optimal pipeline for precise lung cancer diagnosis based on different approaches. The method started with applying a preprocessing process based on a min-max normalization for the input data and an average filter for denoising the input image. Afterward, Kapur entropy maximization along with mathematical morphology was used for segmentation of the lung area. Then, 19 numbers of the GLCM features have been extracted from the segmented images and the features with higher priority were selected based on a new optimization design. The new design, called Improved Thermal Exchange Optimization (ITEO) algorithm, was designed and employed to optimize the feature selection step by considering more accuracy and convergence ability as was shown in the validation stage. Finally, the images were classified into healthy or cancerous cases by using an artificial neural network optimized by ITEO. The simulation was compared with some different state-of-the-art methods including Lin's, Kumar's, and Kavitha's, and the results showed that the proposed method with 92.27% accuracy, 96.4% sensitivity, and 97.61% specificity has the highest efficiency toward the other state-of-the-art methods. In future work, we will work on using convolutional features of the lung cancer images to provide a method with higher accuracy in the system.

## Figures and Tables

**Table 1:** The parameter settings of the studied algorithms.

Algorithm	Parameter	Value	Algorithm	Parameter	Value
SAR [31]	SE	0.5	LSO [28]	F	0.6
	MU	20		L	1
TEO [10]	*c*_1_	0		g	20
	*c*_2_	1	MVO [30]	Traveling distance rate	[0.6, 1]
	pro	0.3		Wormhole existence prob.	[0.2, 1]
	TM	28.5	CSA [29]	fl	2

**Table 2:** The equations and the boundaries of the studied benchmarks in the analysis.

Function	Equation	Constraint
Rastrigin	*f*_1_(*x*)=10 *D*+∑_*i*=1_^*D*^(*x*_*i*_^2^ − 10 cos(2*πx*_*i*_))	[30,50]^*D*^
Rosenbrock	*f*_2_(*x*)=∑_*i*=1_^*D*−1^(100(*x*_*i*_^2^ − *x*_*i*+1_)+(*x*_*i*_ − 1)^2^)	[−2.045, 2.045]^*D*^
Ackley	$f_{3}\left(x\right)=-20\;\exp\left(-0.2\sqrt{\left(1/D\right)\sum_{i=1}^{D}\left(x_{i}^{2}\right)}\right)-\exp\left(\left(1/D\right)\sum_{i=1}^{D}\left(\cos\left(2\pi x_{i}\right)\right)\right)+20+e$	[−10,10]^*D*^
Sphere	*f*_4_(*x*)=∑_*i*=1_^*D*^*x*_*i*_^2^	[−512,512]^*D*^

**Table 3:** The performance analysis of the studied algorithm.

Algorithm		*f*_1_	*f*_2_	*f*_3_	*f*_4_
LSO [28]	Min	11.428	0.035	0.022	1.327
	Max	4.284*e*3	6.534*e*2	0.679*e*5	6.192
	Mean	2.168*e*3	1.295*e*2	2.195*e*4	3.084
	Std	5.153*e*2	21.931	3.645*e*4	4.339

CSA [29]	Min	8.3161	0.028	7.65*e* − 1	1.446
	Max	492.57	2.193	4.15*e* − 1	11.28
	Mean	120.75	1.137	5.17*e* − 1	8.294
	Std	69.253	0.066	1.01*e* − 1	4.495

MVO [30]	Min	9.192	4.168*e* − 3	8.195*e* − 5	0.957
	Max	1.15 *e*2	4.279*e* − 2	7.086*e* − 4	1.492
	Mean	22.49	9.153*e* − 3	1.517*e* − 4	1.137
	Std	38.76	1.097*e* − 3	3.349*e* − 5	0.846

SAR [31]	Min	0.716	4.82*e* − 16	8.16*e* − 7	1.651
	Max	11.05	5.61*e* − 14	2.94*e* − 6	3.287
	Mean	4.13	5.29*e* − 15	1.19*e* − 6	0.201
	Std	3.62	2.46*e* − 14	4.36*e* − 7	0.146

TEO [32]	Min	0.356	9.208*e* − 21	4.192*e* − 9	3.062*e* − 9
	Max	15.349	11.193*e* − 19	5.930*e* − 8	9.836*e* − 8
	Mean	9.3462	34.255*e* − 19	3.591*e* − 8	3.491*e* − 8
	Std	2.896	3.673*e* − 19	5.086*e* − 9	2.038*e* − 9

ITEO	Min	0.00	8.376*e* − 22	6.956*e* − 10	6.763*e* − 13
	Max	4.207	3.205*e* − 21	3.907*e* − 9	6.358*e* − 12
	Mean	3.164	1.343*e* − 21	1.2084*e* − 9	1.928*e* − 12
	Std	1.237	2.934*e* − 21	3.666*e* − 10	1.117*e* − 13

**Table 4:** The GLCM data results of 20 first images from the lung CT-diagnosis database.

Image #	Homogeneity	IMC 1	IMC 2	Inverse difference	Highest probability	Sum average	Sum entropy	Sum of square variance	Sum variance
1	−0.916	−0.914	0.654	0.997	0.717	2.372	0.407	0.553	0.662
2	−0.900	−0.879	0.212	0.992	0.531	2.357	0.552	0.506	0.711
3	−0.884	−0.88	0.625	0.991	0.891	2.68	0.69	0.147	0.374
4	−0.891	−0.929	0.476	0.992	0.529	2.472	0.601	0.147	0.21
5	−0.911	−0.903	0.476	0.992	0.692	2.517	0.558	0.211	0.091
6	−0.895	−0.915	0.274	0.993	0.865	2.532	0.23	0.123	0.075
7	−0.915	−0.875	0.713	0.993	0.674	2.308	0.384	0.397	0.43
8	−0.882	−0.914	0.405	0.991	0.771	2.41	0.495	0.659	0.554
9	−0.881	−0.859	0.355	0.995	0.861	2.434	0.423	0.517	0.078
10	−0.888	−0.866	0.416	0.992	0.891	2.194	0.198	0.453	0.483
11	−0.917	−0.905	0.437	0.992	0.911	2.437	0.296	0.68	0.382
12	−0.913	−0.895	0.544	0.995	0.584	2.517	0.629	0.174	0.14
13	−0.925	−0.877	0.554	0.997	0.614	2.356	0.179	0.709	0.677
14	−0.893	−0.865	0.449	0.998	0.896	2.602	0.645	0.181	0.433
15	−0.887	−0.885	0.451	0.995	0.762	2.539	0.213	0.451	0.328
16	−0.885	−0.888	0.525	0.998	0.722	2.316	0.538	0.204	0.679
17	−0.907	−0.911	0.614	0.995	0.77	2.373	0.445	0.479	0.479
18	−0.904	−0.899	0.799	0.995	0.861	2.332	0.29	0.113	0.336
19	−0.931	−0.876	0.655	0.993	0.735	2.565	0.484	0.616	0.068
20	−0.886	−0.86	0.452	0.994	0.589	2.541	0.237	0.67	0.393

**Table 5:** The GLCM data results of 20 first images from the lung CT-diagnosis database.

Image #	Autocorrelation	Cluster prominence	Cluster shade	Contrast	Correlation	Difference entropy	Difference variance	Dissimilarity	Energy	Entropy
1	1.5292	0.9281	0.2484	0.0031	0.951	0.0101	0.001	0.001	0.8911	0.2308
2	1.7732	1.4425	0.5614	0.0049	0.9617	0.017	0.003	0.0029	0.749	0.424
3	1.5332	0.9471	0.2584	0.0024	0.9637	0.0023	0.0005	0.0004	0.8903	0.2274
4	2.0352	1.5474	0.6374	0.0086	0.957	0.0357	0.0063	0.0066	0.6421	0.5764
5	1.4202	0.5069	0.0204	0.0031	0.9247	0.0045	0.001	0.002	0.9595	0.101
6	1.3862	0.3493	0.1646	0.0015	0.9515	0.0133	0.0014	0.0014	0.984	0.0444
7	0.7428	0.7435	0.3716	0.0035	0.9413	0.029	0.0033	0.0035	0.9226	0.1792
8	0.7528	0.8409	0.4256	0.0024	0.975	0.0352	0.0046	0.0044	0.9064	0.2137
9	0.7668	0.8365	0.4216	0.0007	0.994	0.0257	0.003	0.0029	0.9105	0.2015
10	1.4782	0.7423	0.3716	0.0029	0.9603	0.0381	0.0051	0.005	0.921	0.1889
11	1.5302	0.9288	0.4746	0.0024	0.9826	0.0353	0.0045	0.0046	0.891	0.2391
12	1.5432	0.9597	0.2674	0.0039	0.9666	0.0466	0.006	0.005	0.881	0.2612
13	1.7702	1.4266	0.5524	0.0057	0.9891	0.0522	0.0077	0.0058	0.7585	0.4381
14	1.7072	0.8725	0.5024	0.0071	0.9858	0.0315	0.005	0.004	0.784	0.3995
15	1.0348	0.9531	0.7806	0.0072	0.9916	0.0294	0.0052	0.0051	0.756	0.4349
16	0.8828	0.6969	0.6166	0.0038	0.9594	0.0109	0.0018	0.0017	0.8405	0.3082
17	0.8948	0.7077	0.6256	0.0091	0.9283	0.0383	0.009	0.0069	0.8264	0.3453
18	2.0578	1.2224	0.1826	0.0204	0.94	0.105	0.0199	0.02	0.4803	0.7899
19	0.6988	0.5715	0.2796	0.002	0.955	0.0184	0.002	0.0019	0.9541	0.1186
20	1.7448	1.3354	0.5706	0.0141	0.9508	0.0811	0.0141	0.014	0.5192	0.7325

**Table 6:** The optimum features using the proposed ITEO applied to GLCM.

Image #	Autocorrelation	Correlation	Energy	Homogeneity	Inverse difference	Highest probability	Sum average	Class
1	1.163	0.97	0.8911	0.9972	0.9971	0.9417	2.1095	Yes
2	1.407	0.9807	0.749	0.9966	0.9965	0.8594	2.2728	Yes
3	1.167	0.9827	0.8903	0.9979	0.9978	0.9411	2.1119	Yes
4	1.669	0.976	0.6421	0.9948	0.9947	0.769	2.4488	Yes
5	1.054	0.9437	0.9595	0.997	0.997	0.969	2.036	Yes
6	1.02	0.9705	0.986	0.9985	0.9984	0.9931	2.0131	Yes
7	1.111	0.9603	0.9246	0.9993	0.9972	0.9611	2.0748	Yes
8	1.121	0.954	0.9064	0.9988	0.9987	0.9512	2.0955	Yes
9	1.135	0.975	0.9105	0.9996	0.9995	0.9534	2.0928	Yes
10	1.112	0.9413	0.921	0.9985	0.9984	0.959	2.0782	Yes
11	1.164	0.9636	0.891	0.997	0.998	0.94	2.1127	Yes
12	1.177	0.9476	0.879	0.996	0.997	0.9351	2.121	Yes
13	1.404	0.9701	0.7565	0.9971	0.995	0.859	2.2727	Yes
14	1.341	0.9648	0.784	0.9952	0.9951	0.8774	2.2339	No
15	1.403	0.9706	0.756	0.9954	0.9953	0.8585	2.2723	No
16	1.251	0.9784	0.8405	0.9971	0.997	0.9142	2.1682	No
17	1.263	0.9473	0.8284	0.9945	0.9944	0.9062	2.1787	No
18	2.426	0.961	0.4823	0.9907	0.9907	0.5114	2.9592	No
19	1.067	0.976	0.9541	1.0001	1	0.9767	2.0473	No
20	2.113	0.9718	0.5192	0.994	0.9939	0.6199	2.7485	No

## Data Availability

The data that support the findings will be available in the Lung CT-Diagnosis database of lung cancer images at https://doi.org/10.7937/K9/TCIA.2015.A6V7JIWX.
